# Supplementary material for: Topological data analysis in air traffic management: The shape of big flight data sets
Source: PLoS One. 2025 Feb 27;20(2):e0318108. doi: 10.1371/journal.pone.0318108 (PMC11867395; doi:10.1371/journal.pone.0318108)
Supplement: S1 Text — (PDF) S2 Text: Statistical significance: p-values. (PDF) S3 Text: Centrality measures. (PDF) [file pone.0318108.s001.pdf]

Supporting information

S1 Text: Tables

	LEAB	LEBZ	LELN	LESA	LESB	LEVD
LEAB	0.00	106.28	58.60	129.64	112.24	107.70
LEBZ	106.28	0.00	53.22	54.47	23.73	42.73
LELN	58.60	53.22	0.00	100.65	69.99	49.32
LESA	129.64	54.47	100.65	0.00	38.71	74.42
LESB	112.24	23.73	69.99	38.71	0.00	61.62
LEVD	107.70	42.73	49.32	74.42	61.62	0.00

Table 1. Distance matrix corresponding to Group 3 - *Air bases open to civilian traffic*

	LEAB	LEBZ	LELN	LESA	LESB	LEVD
LECU	123.04	34.12	85.25	27.17	16.24	59.85
LELL	162.93	75.88	125.81	35.15	57.94	94.85

Table 2. Distance matrix corresponding to Group 3 - *Air bases open to civilian traffic* and the Group 3 - *General aviation airports*

	LEAB	LEBZ	LELN	LESA	LESB	LEVD
GEML	181.95	118.74	160.00	64.59	102.96	138.88
LEBA	119.56	32.91	81.47	33.18	14.24	72.80
LEBG	144.18	58.86	109.44	16.39	40.72	83.15
LEGE	77.39	46.06	46.60	65.33	59.18	36.74
LEHC	126.42	83.75	115.76	32.04	69.63	103.73
LEPP	102.69	17.30	56.70	43.97	34.28	34.95
LERJ	126.29	58.37	102.48	15.52	41.34	78.15
LESO	132.98	56.64	102.03	30.05	39.73	76.50
LEVT	99.37	38.94	60.45	60.09	53.85	32.98

Table 3. Distance matrix corresponding to Group 3 - *Air bases open to civilian traffic* and the Group 3 - *Airports with low traffic*

	LEAB	LEBZ	LELN	LESA	LESB	LEVD
LEAM	112.78	48.08	72.95	67.41	62.13	35.08
LEAS	118.22	39.16	75.56	57.19	53.91	42.99
LECO	139.56	39.85	93.06	44.68	42.57	54.90
LEGR	131.81	39.66	88.52	55.22	52.58	49.40
LEJR	98.17	42.70	52.22	61.72	57.50	26.37
LERS	109.29	48.25	58.51	68.06	62.81	25.06
LEVX	120.37	35.99	77.80	50.96	49.25	41.39
LEXJ	118.34	41.27	69.07	56.62	55.20	30.72
LEZG	125.63	189.13	143.81	192.98	189.22	191.02

Table 4. Distance matrix corresponding to Group 3 - *Air bases open to civilian traffic* and the Group 2

	LEAB	LEBZ	LELN	LESA	LESB	LEVD
LEAL	47.75	66.99	35.10	82.37	67.93	69.30
LEBB	84.02	36.38	50.21	50.66	46.71	37.36
LEIB	84.27	42.43	47.18	58.95	46.47	44.49
LEMG	41.04	104.21	57.36	119.10	105.47	106.33
LEMH	119.76	37.16	69.92	53.32	50.55	31.70
LEST	102.49	42.78	59.97	56.79	54.88	27.46
LEVC	55.67	54.35	34.99	76.36	56.65	56.30
LEZL	87.26	36.69	46.76	55.46	47.77	29.80

Table 5. Distance matrix corresponding to Group 3 - *Air bases open to civilian traffic* and the Group 1

	LEAB	LEBZ	LELN	LESA	LESB	LEVD
GCFV	46.31	78.20	46.51	98.36	81.18	80.42
GCGM	182.02	122.09	161.68	68.55	107.16	142.10
GCHI	184.57	124.98	164.57	71.30	109.97	145.08
GCLA	51.30	57.61	38.93	100.42	71.33	56.45
GCLP	45.91	79.78	48.25	86.12	82.01	79.44
GCRR	50.11	63.25	35.79	82.53	63.65	65.42
GCTS	88.64	143.24	101.00	142.02	144.74	143.84
GCXO	112.29	34.75	73.34	38.31	39.44	44.84

Table 6. Distance matrix corresponding to Group 3 - *Air bases open to civilian traffic* and the Canary Group

	LEAB	LEBZ	LELN	LESA	LESB	LEVD
LEBL	306.99	353.02	315.33	343.49	356.43	350.35
LEMD	453.15	462.99	457.91	460.78	465.56	462.91
LEPA	72.94	33.36	30.60	70.09	44.31	35.09

Table 7. Distance matrix corresponding to Group 3 - *Air bases open to civilian traffic* and the Special Group

	LECU	LELL
LECU	0.00	41.91
LELL	41.91	0.00

Table 8. Distance matrix corresponding to Group 3 - *General aviation airports*

	GEML	LEBA	LEBG	LEGE	LEHC	LEPP	LERJ	LESO	LEVT
LECU	89.06	21.01	28.24	54.45	59.20	30.10	26.73	23.76	48.71
LELL	47.78	47.37	21.46	85.80	64.00	69.90	50.25	32.80	67.38

Table 9. Distance matrix corresponding to Group 3 - *General aviation airports* and Group 3 - *Airports with low traffic*

	LEAM	LEAS	LECO	LEGR	LEJR	LERS	LEVX	LEXJ	LEZG
LECU	57.34	48.19	34.79	46.54	52.02	58.10	42.28	49.27	191.09
LELL	74.92	63.95	49.94	61.65	75.69	75.95	56.54	66.50	227.78

Table 10. Distance matrix corresponding to Group 3 - *General aviation airports* and Group 2

	LEAL	LEBB	LEIB	LEMG	LEMH	LEST	LEVC	LEZL
LECU	76.43	40.00	44.15	113.35	44.85	48.73	68.11	41.42
LELL	116.14	79.10	81.42	152.04	65.81	67.53	108.09	79.07

Table 11. Distance matrix corresponding to Group 3 - *General aviation airports* and Group 1

	GCFV	GCGM	GCHI	GCLA	GCLP	GCRR	GCTS	GCXO
LECU	91.67	94.84	97.42	85.22	80.52	73.31	139.60	27.44
LELL	131.56	59.15	60.76	124.78	119.50	113.23	176.94	52.46

Table 12. Distance matrix corresponding to Group 3 - *General aviation airports* and the Canary Group

	LEBL	LEMD	LEPA
LECU	344.80	459.58	56.15
LELL	367.96	465.56	96.01

Table 13. Distance matrix corresponding to Group 3 - *General aviation airports* and the Special Group

	LEAM	LEAS	LECO	LEGR	LEJR	LERS	LEVX	LEXJ	LEZG
GCFV	81.48	88.23	108.45	100.62	67.72	80.53	89.26	87.79	117.50
GCGM	122.74	109.65	101.75	110.15	124.66	121.86	106.46	119.41	242.30
GCHI	125.27	111.88	104.02	112.59	127.40	124.45	109.13	122.06	244.70
GCLA	74.32	75.58	96.38	90.27	53.69	61.72	79.79	73.31	149.05
GCLP	72.40	85.90	100.04	92.57	70.58	78.18	79.96	80.55	112.87
GCRR	63.34	70.80	90.06	82.17	49.86	63.53	70.86	69.64	127.64
GCTS	129.36	149.25	161.29	153.26	131.04	142.48	140.05	143.84	61.07
GCXO	41.27	40.97	45.53	34.54	33.13	42.15	30.55	34.00	177.50

Table 14. Distance matrix corresponding to Group 2 and the Canary Group

	LEAM	LEAS	LECO	LEGR	LEJR	LERS	LEVX	LEXJ	LEZG
LEBL	345.30	360.04	365.06	354.35	343.12	349.85	348.75	351.07	185.01
LEMD	465.38	465.56	465.56	461.14	464.49	464.01	463.71	465.56	378.29
LEPA	45.21	46.44	68.64	61.62	26.45	37.20	50.77	46.78	160.32

Table 15. Distance matrix corresponding to Group 2 and the Special Group

	LEAL	LEBB	LEIB	LEMG	LEMH	LEST	LEVC	LEZL
GCFV	26.65	52.65	52.89	26.04	90.16	71.77	27.20	55.99
GCGM	139.67	117.15	126.72	174.18	117.29	118.14	131.50	120.68
GCHI	142.64	119.99	129.74	176.85	120.03	120.86	134.19	123.73
GCLA	23.48	50.21	46.73	55.59	73.78	60.72	33.89	46.31
GCLP	43.73	57.89	48.47	33.18	87.13	65.61	46.17	60.46
GCRR	12.96	34.19	34.61	42.75	72.65	53.39	15.37	37.72
GCTS	89.16	108.25	102.16	62.74	150.96	129.57	97.67	115.63
GCXO	65.62	28.50	31.22	101.74	41.14	27.03	57.35	30.71

Table 16. Distance matrix corresponding to Group 1 and the Canary Group

	GCFV	GCGM	GCHI	GCLA	GCLP	GCRR	GCTS	GCXO
LEBL	296.47	366.86	367.96	327.56	274.82	306.85	219.22	319.87
LEMD	448.45	465.56	465.56	463.43	419.33	460.48	436.09	449.87
LEPA	46.63	132.85	135.83	30.78	67.96	34.24	119.95	43.95

Table 17. Distance matrix corresponding to the Canary Group and the Special Group

S2 Text: Statistical significance: p-values

	LECU	LELL	LEAB	LEBZ	LELN	LESA	LESB	LEVD	GEML	LEBG	LEBA	LEGE	LEHC	LERJ	LEPP	LESO	LEVT
LECU						0.0675	0.5575			0.1025	0.68			0.13		0.08	
LELL										0.085							
LEAB																	
LEBZ							0.1875				0.255				0.3425		
LELN																	
LESA	0.0675									0.56	0.2225		0.3575	0.6925			
LESB	0.5575			0.1875							0.8825						
LEVD																	
GEML																	
LEBG	0.1025	0.085				0.56					0.1975		0.1	0.155			
LEBA	0.68			0.255		0.2225	0.8825			0.1975				0.29	0.06	0.1725	
LEGE												0.1225				0.25	
LEHC						0.3575				0.1		0.1225		0.37	0.18	0.18	
LERJ	0.13					0.6925				0.155	0.29		0.37				
LEPP				0.3425							0.06		0.18				0.155
LESO	0.08										0.1725		0.18				
LEVT												0.25			0.155		
LECO																	
LEAM																	0.505
LEAS																	0.18
LEGR																	
LEJR								0.08				0.2225					0.6125
LERS																	0.5025
LEXJ													0.145				0.3125
LEST								0.065				0.1475			0.0875		0.8
LEVX															0.13		0.2575
LEZG																	
LEAL			0.185		0.1025							0.15					
LEBB											0.08	0.25	0.2475		0.065		0.38
LEIB											0.2575	0.415	0.245				0.1925
LEMG			0.3525														
LEMH															0.0975		0.16
LEZL								0.135			0.065	0.6775	0.2275		0.2275		0.5825
LEVC			0.0825		0.1025							0.375					
GCFV			0.2225														
GCGM																	
GCHI																	
GCLA			0.0775														
GCLP			0.325														
GCRR			0.1025		0.1075							0.1175					
GCTS													0.33				
GCXO	0.1125										0.21	0.0525	0.1575		0.13	0.0625	0.13
LEMD													0.2375				
LEBL																	
LEPA					0.14						0.0875	0.2725	0.12				0.07

Table 18. p-values between Group 3 and the rest of the airports

	LECO	LEAM	LEAS	LEGR	LEJR	LERS	LEXJ	LEST	LEVX	LEZG	LEAL	LEBB	LEIB	LEMG	LEMH	LEZL	LEVC
LECU																	
LELL																	
LEAB											0.185			0.3525			0.0825
LEBZ																	
LELN											0.1025						0.1025
LESA																	
LESB																	
LEVD					0.08			0.065								0.135	
GEML																	
LEBG																	
LEBA												0.08	0.2575			0.065	
LEGE					0.2225			0.1475			0.15	0.25	0.415			0.6775	0.375
LEHC							0.145					0.2475	0.245			0.2275	
LERJ																	
LEPP								0.0875	0.13			0.065			0.0975	0.2275	
LESO																	
LEVT		0.505	0.18		0.6125	0.5025	0.3125	0.8	0.2575			0.38	0.1925		0.16	0.5825	
LECO			0.075	0.2725			0.0775		0.1325								
LEAM			0.1875	0.055	0.275	0.3925	0.425	0.3125				0.1	0.0875		0.1325	0.14	
LEAS	0.075	0.1875		0.31	0.105	0.18	0.4075	0.1225	0.575						0.42		
LEGR	0.2725	0.055	0.31				0.1575		0.52						0.1575		
LEJR		0.275	0.105			0.5175	0.2275	0.4625	0.0575			0.2725	0.1325		0.14	0.6275	
LERS		0.3925	0.18		0.5175		0.3075	0.2325							0.225	0.1375	
LEXJ	0.0775	0.425	0.4075	0.1575	0.2275	0.3075		0.415	0.3625						0.7725	0.08	
LEST		0.3125	0.1225		0.4625	0.2325	0.415		0.285			0.2425	0.12		0.115	0.2775	
LEVX	0.1325		0.575	0.52	0.0575		0.3625	0.285							0.17	0.0725	
LEZG																	
LEAL												0.085	0.13	0.1125			0.6825
LEBB		0.1			0.2725			0.2425			0.085		0.61			0.2675	0.19
LEIB		0.0875			0.1325			0.12			0.13	0.61				0.5175	0.2075
LEMG											0.1125						
LEMH		0.1325	0.42	0.1575	0.14	0.225	0.7725	0.115	0.17								
LEZL		0.14			0.6275	0.1375	0.08	0.2775	0.0725			0.2675	0.5175				0.145
LEVC											0.6825	0.19	0.2075			0.145	
GCFV											0.3325			0.415			0.2875
GCGM																	
GCHI																	
GCLA											0.3625						0.14
GCLP											0.0625		0.0575	0.2675			
GCRR											0.8525	0.12	0.1			0.0825	0.7325
GCTS										0.095							
GCXO					0.0875			0.2375	0.1			0.245	0.235			0.1875	
LEMD																	
LEBL																	
LEPA					0.1075						0.0675	0.3575	0.3325			0.6025	0.3075

Table 19. p-values between Group 2 and Group 1 and the rest of the airports

	GCFV	GCGM	GCHI	GCLA	GCLP	GCRR	GCTS	GCXO	LEMD	LEBL	LEPA
LECU								0.1125			
LELL											
LEAB	0.2225			0.0775	0.325	0.1025					
LEBZ											
LELN						0.1075					0.14
LESA											
LESB											
LEVD											
GEML											
LEBG											
LEBA								0.21			0.0875
LEGE						0.1175		0.0525			0.2725
LEHC							0.33	0.1575	0.2375		
LERJ											
LEPP								0.13			0.075
LESO								0.0625			
LEVT								0.13			0.07
LECO											
LEAM											
LEAS											
LEGR											
LEJR								0.0875			0.1075
LERS											
LEXJ											
LEST								0.2375			0.06
LEVX								0.1			
LEZG							0.095				
LEAL	0.3325			0.3625	0.0625	0.8525					0.0675
LEBB						0.12		0.245			0.3575
LEIB					0.0575	0.1		0.235			0.3325
LEMG	0.415				0.2675						
LEMH											
LEZL								0.1875			0.6025
LEVC	0.2875			0.14		0.7325					0.3075
GCFV				0.215	0.145	0.3025					
GCGM											
GCHI											
GCLA	0.215					0.3925					0.14
GCLP	0.145										
GCRR	0.3025			0.3925							0.0825
GCTS											
GCXO											
LEMD											
LEBL											
LEPA				0.14		0.0825					

Table 20. p-values between Canary Group and Special Group and the rest of the airports

S3 Text: Centrality measures

	Betweenness	*Degree*	*Closeness*	*Local reaching*
LEMD	0.0228	0.2736	0.1659	0.0227
LEBL	0.0176	0.2606	0.163	0.0175
LEPA	0.0113	0.1866	0.1457	0.0113
LEMG	0.0064	0.1589	0.1401	0.0064
LEAL	0.0043	0.124	0.1321	0.0043
GCLP	0.0037	0.0965	0.1267	0.0037
LEIB	0.0031	0.0961	0.1296	0.0031
LEVC	0.0019	0.0886	0.1286	0.0019
GCTS	0.0016	0.0844	0.1198	0.0016
LEGE	0.0016	0.0462	0.0958	0.0016
LEZL	0.0011	0.0677	0.1248	0.0011
LEZG	0.001	0.0203	0.0983	0.001
GCRR	0.0007	0.0557	0.1172	0.0007
LEMH	0.0007	0.0416	0.1192	0.0007
GCXO	0.0007	0.0249	0.1166	0.0007
GCFV	0.0006	0.0532	0.1164	0.0006
LEBB	0.0004	0.053	0.1256	0.0004
LEST	0.0003	0.0302	0.1196	0.0003
LEVD	0.0003	0.006	0.1005	0.0003
LECU	0.0002	0.0049	0.0731	0.0003
LERS	0.0002	0.0207	0.0876	0.0002
LEJR	0.0002	0.0195	0.1106	0.0002
LEAM	0.0002	0.0182	0.1108	0.0002
LESO	0.0002	0.0053	0.106	0.0002
LEVX	0.0002	0.0117	0.1084	0.0002
LEGR	0.0002	0.0123	0.1139	0.0002
LEPP	0.0002	0.0048	0.1012	0.0002
LEAS	0.0002	0.0185	0.1167	0.0002
LEXJ	0.0001	0.0154	0.1115	0.0001
LEVT	0.0001	0.0152	0.0962	0.0001
LECO	0.0001	0.0113	0.1096	0.0001
LELL	0.0001	0.0022	0.0303	0.0001
LELN	0.0001	0.0031	0.0842	0.0001
LESA	0.0001	0.0035	0.0563	0.0001
LEAB	0.0001	0.0011	0.0179	0.0001
LEBZ	0.0001	0.0032	0.0904	0.0001
LESB	0.0001	0.0014	0.022	0.0001
LERJ	0.0	0.0015	0.0709	0.0
GCLA	0.0	0.0101	0.1045	0.0
GEML	0.0	0.0039	0.1048	0.0
LEBG	0.0	0.0008	0.0374	0.0
LEHC	0.0	0.0004	0.0088	0.0
LEBA	0.0	0.0006	0.0191	0.0
GCGM	0.0	0.0021	0.08	0.0
GCHI	0.0	0.0033	0.0846	0.0

Table 21
